# Exploring metabolic anomalies in COVID-19 and post-COVID-19: a machine learning approach with explainable artificial intelligence

**DOI:** 10.3389/fmolb.2024.1429281

**Published:** 2024-09-09

**Authors:** Juan José Oropeza-Valdez, Cristian Padron-Manrique, Aarón Vázquez-Jiménez, Xavier Soberon, Osbaldo Resendis-Antonio

**Affiliations:** ^1^ Human Systems Biology Laboratory. Instituto Nacional de Medicina Genómica (INMEGEN), Mexico City, Mexico; ^2^ Centro de Ciencias de la Complejidad, Universidad Nacional Autónoma de México (UNAM), Mexico City, Mexico; ^3^ Programa de Doctorado en Ciencias Biomédicas, Universidad Nacional Autónoma de México (UNAM), Mexico City, Mexico; ^4^ Departamento de Ingeniería Celular y Biocatálisis, Instituto de Biotecnología, Universidad Nacional Autónoma de México (UNAM), Colonia Chamilpa, Cuernavaca, México; ^5^ Coordinación de la Investigación Científica – Red de Apoyo a la Investigación, Universidad Nacional Autónoma de México (UNAM), Mexico City, Mexico

**Keywords:** metabolomics, explainable artificial intelligence (XAI), COVID-19, post-COVID-19, machine learning (ML), long Covid

## Abstract

The COVID-19 pandemic, caused by SARS-CoV-2, has led to significant challenges worldwide, including diverse clinical outcomes and prolonged post-recovery symptoms known as Long COVID or Post-COVID-19 syndrome. Emerging evidence suggests a crucial role of metabolic reprogramming in the infection’s long-term consequences. This study employs a novel approach utilizing machine learning (ML) and explainable artificial intelligence (XAI) to analyze metabolic alterations in COVID-19 and Post-COVID-19 patients. Samples were taken from a cohort of 142 COVID-19, 48 Post-COVID-19, and 38 control patients, comprising 111 identified metabolites. Traditional analysis methods, like PCA and PLS-DA, were compared with ML techniques, particularly eXtreme Gradient Boosting (XGBoost) enhanced by SHAP (SHapley Additive exPlanations) values for explainability. XGBoost, combined with SHAP, outperformed traditional methods, demonstrating superior predictive performance and providing new insights into the metabolic basis of the disease’s progression and aftermath. The analysis revealed metabolomic subgroups within the COVID-19 and Post-COVID-19 conditions, suggesting heterogeneous metabolic responses to the infection and its long-term impacts. Key metabolic signatures in Post-COVID-19 include taurine, glutamine, alpha-Ketoglutaric acid, and LysoPC a C16:0. This study highlights the potential of integrating ML and XAI for a fine-grained description in metabolomics research, offering a more detailed understanding of metabolic anomalies in COVID-19 and Post-COVID-19 conditions.

## Introduction

The COVID-19 pandemic, caused by the coronavirus SARS-CoV-2, has presented a formidable challenge to global health systems. As of March 2024, the number of confirmed COVID-19 cases has surpassed 770 million (WHO Coronavirus). The wide spectrum of symptoms, varying from mild to severe respiratory distress and multi-organ dysfunction (Zhao et al., 2022), underscores the need for a comprehensive systemic understanding of the disease’s pathophysiology and the factors contributing to its diverse clinical outcomes (Al Sulaiman et al., 2023; Reyes et al., 2022). In addition to the immediate health impacts, the COVID-19 pandemic has highlighted the long-lasting effects and challenges in the post-recovery phase. Many individuals who have recovered from COVID-19 have reported a wide range of persistent symptoms and health issues (Khodeir et al., 2021; Galván-Tejada et al., 2020). Common symptoms following recovery include persistent fatigue, shortness of breath, cough, joint and chest pain, brain fog, depression, and anxiety (Phetsouphanh et al., 2022; CDC, 2023). Moreover, the full extent of these symptoms and their long-term consequences remain uncharacterized. The post-recovery symptoms, often referred to as “Post-COVID-19”or “Long COVID-19”syndrome, can persist for weeks or up to 2 years after the initial infection (Ballouz et al., 2023). Although certain mechanisms, viral persistence (Chen B. et al., 2023), immune dysregulation (Phetsouphanh et al., 2022), and organ damage (Iqbal et al., 2023), have been identified as potentially involved in Post-COVID-19 symptoms, their exact understanding remains incomplete. One emblematic factor accompanying the post-symptoms is metabolic reprogramming at the systemic level. Emerging evidence suggests the long-term consequences of COVID-19 may be linked to systemic metabolic reprogramming during infection, affecting pathways related to amino acids, glucose, cholesterol, fatty acids, among others (Chen P. et al., 2023). This metabolic disruption alters energy production and immune regulation, pointing to a need for further research to understand these changes and develop specific therapeutic interventions.

Metabolomics offers a comprehensive and unbiased view of the biochemical alterations occurring during viral infections, portraying the complex interactions between the viral pathogen and the host response (Manchester and Anisha Anand, 2017; Palmer, 2022). Notably, this approach has been proven successful in uncovering distinct metabolic signatures associated with various infectious diseases (Rahman and Schellhorn, 2023), including COVID-19 and Post-COVID-19.

Under statistical-based approaches, several studies have contributed to characterizing the convoluted metabolic changes across COVID-19 progression over the diverse SARS-CoV-2 variants; severity and morbidity markers have been identified related to the progression to the immune over activation, particularly the relation of tryptophan and Kynurenine, the transformation of L-tryptophan, and the rise of the levels of taurochenodeoxycholic, propylparaben, 20-hydroxyeicosatetraenoic acid, acid 3-sulfate, and glucuronate (Thomas et al., 2020; Chen et al., 2021; Mangge et al., 2021; Lawler et al., 2021; Kimhofer et al., 2020; Ceballos et al., 2022; Li et al., 2023; Abdallah et al., 2024; Cyprian et al., 2023). Several plasma pro-inflammatory biomarkers showed a significant correlation with deregulated metabolites and metabolic signatures (Chen P. et al., 2023; López-Hernández et al., 2021; Martínez-Gómez et al., 2022; Shen et al., 2020; Pang et al., 2021a; Ghini et al., 2023). Post-COVID-19 metabolic characterization showed a relation between the symptomatology and increased levels of several species of phosphatidylcholines and sphingomyelins (López-Hernández et al., 2023a). In addition, the leukocyte metabolism is altered, affecting long-lasting immunity, dyslipidemia, and energy metabolism dysregulation; there is a decrease in the cortisol and metabolites of mitochondrial dysfunction (Tsilingiris et al., 2023; Fanelli et al., 2024; Ansone et al., 2024). Contrastingly, some reports show a normalization in the metabolic levels as the infection clears out (López-Hernández et al., 2023a; Liptak et al., 2022). Despite the valuable endeavors, metabolome characterization is hidden under tangled layers of information with high dimensionality and nonlinear interaction nature (Tebani et al., 2018).

Traditionally, linear dimensionality reduction methods are used to identify low-dimensional embedding spaces in metabolomic data. Among these methods, PCA (Principal Component Analysis) and its supervised counterpart the PLS-DA (Partial Least Square Discriminant Analysis) (Ruiz-Perez et al., 2020) are the most frequent. Despite their importance, these methods exhibit significant limitations when it comes to uncovering and analyzing nonlinear interactions, which are often crucial in differentiating intricate groups, such as control versus disease phenotype (Shiokawa et al., 2018). Alternatively, differential expression analysis applied in metabolic concentrations is a well-established technique to identify metabolites with significant statistical differences expressed between or among clinical groups. This latter strategy detects the over-representation of features within a class identified by the magnitudes of these changes using p-values. As a result, it falls short in detecting complex interactions. To overcome this limitation, some non-supervised and supervised machine learning algorithms have been suggested to take into account the linear and non-linear interactions emerging from metabolome data. For instance, Uniform Manifold Approximation and Projection (UMAP), an unsupervised reduction method in multidimensional data, captures the complex topology of high-dimensional spaces and effectively reduces it to a lower-dimensional representation. This approach provides superior projections and enhanced cluster separation in handling intricate data structures compared to other dimensional reduction methods such as PCA, t-SNE, or autoencoders (McInnes et al., 2018). However, features with low variable magnitude typically have a reduced impact on these low-dimensional projections due to their dependence on distance metrics, even though they can be informative for phenotype classification. Furthermore, supervised machine learning algorithms, like eXtreme Gradient Boosting (XGBoost) (Chen and Guestrin, 2016), have emerged as a solution to identify those variables that play an important role in classifying groups of multidimensional data. This classification algorithm is insensitive to feature magnitude variations, capable of discerning subtle and or complex patterns, transcending the limitations of traditional methods and over-representation biases. The insensitivity of XGBoost to feature magnitude means that it does not require extensive data preprocessing to normalize or to scale the features, making it more robust and easier to apply due to its tree-based method. Notably, by combining this approach with the SHAP (SHapley Additive exPlanations) method, XGBoost goes beyond detecting the high and low magnitudes of metabolites to classify a phenotype.

Particularly, SHAP values are a tool used in Explainable Artificial Intelligence (XAI) to interpret machine learning models by showing how much each feature (metabolite) contributes to the model’s final prediction (phenotype class, like healthy or COVID). Drawing from game theory, SHAP values treat each feature as a player in a game, contributing to the outcome. They calculate the importance of each feature by adding and removing features for each instance (sample) and observing changes in the prediction. This process, done across all possible feature combinations and instances, determines the individual impact of each feature on the prediction (Lundberg and Lee, 2017). In the end, a SHAP value matrix is generated where each row represents an instance from the dataset, and each column represents a feature. The values within the matrix show the contribution of each feature to the prediction for each instance (Lundberg et al., 2020). SHAP values can be used to rank the importance of each feature in making predictions (global explainability) by averaging their contributions across all instances or to elucidate how individual predictions are derived (local explainability) by showing the contribution of each feature for a specific instance. This helps us understand how each metabolite influences the model’s decision for each sample and overall (Lundberg et al., 2020).

Interestingly, SHAP matrix (*a.k.a.* local explainability) can be employed for supervised clustering to create explainable embeddings (Lundberg et al., 2020). Thus, for a metabolome dataset, each sample’s multidimensional metabolic profile can be represented in a reduced dimensional space while preserving the explainability of individual features for the prediction (Lundberg and Lee, 2017). As we show in this paper, these explainable embedding spaces are unbiased by the magnitude or scale of the variables when we use XGBoost (Filho, 2023; Is Normalization necessary). In this type of explainable embedding, similarities between samples are determined by the importance of the weight for classification rather than the original values (Chen and Guestrin, 2016; Lundberg et al., 2020). While explainable embeddings have been employed in metabolomics (Bifarin, 2023), they have never been used before in COVID-19 or Post-COVID-19 studies to the best of our knowledge. Therefore, there is a need to use these new approaches to identify novel groups related to Post-COVID-19, particularly in areas where traditional unsupervised methods reach their limits.

In the context of understanding metabolic anomalies between COVID-19 and Post-COVID-19 phenotypes, the objective of the study is to contrast the biomarkers obtained from previous studies already published (López-Hernández et al., 2021; López-Hernández et al., 2023b) with advanced machine learning algorithms combined with analyses of global and local explainability. Altogether, allowed us to conduct a detailed and multifaceted exploration of metabolites distinguishing both phenotypes. Our analysis not only suggests potential biomarkers through differential expression analysis but also contributes to the understanding of metabolic alterations by combining machine learning and Explainable Artificial Intelligence (XAI).

## Results

### Overview of the analysis and cohort study

**FIGURE 1:**
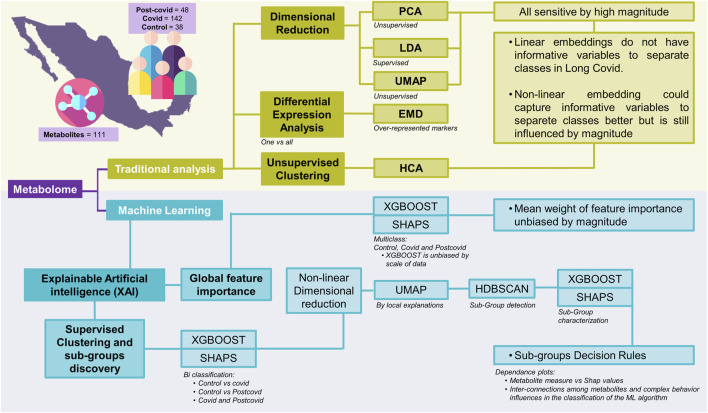
Schematic representation of various analytical approaches applied to the integrated metabolome data. The acronyms and their respective meanings are: PCA: Principal Component Analysis - An unsupervised method that transforms the original variables into a new set of variables called principal components. PLS-DA: Partial Least Squares Discriminant Analysis - A supervised technique that seeks to find a linear combination of features that best separates two or more classes in a dataset. UMAP: Uniform Manifold Approximation and Projection - A non-linear dimensionality reduction technique that works well for clustering and visual representation of high-dimensional datasets. EMD: Earth Mover’s Distance - A measure of the distance between two probability distributions, conceptualized as the minimum “work” needed to transform one distribution into another. It is also employed as a measure for differential expression by clusters. HCA: Hierarchical Clustering Analysis - A method of cluster analysis which seeks to build a hierarchy of clusters. XAI: Explainable Artificial Intelligence - A branch of AI that aims to make the decision-making process of machine learning models transparent and understandable. XGBOOST: Extreme Gradient Boosting - A highly efficient and scalable implementation of gradient boosting that works for both regression and classification problems. RF: Random Forest - An ensemble method that builds multiple decision trees for robust classification and regression outputs. SVM: Support Vector Machine - A powerful classifier that finds the optimal hyperplane for categorizing data into two distinct classes. LogReg: Logistic Regression - A statistical model that estimates probabilities of binary outcomes based on input features, adaptable to multiclass problems. SHAPS: SHapley Additive exPlanations - A method to explain individual predictions of any machine learning model by computing the contribution of each feature to every prediction. HDBSCAN: Hierarchical Density-Based Spatial Clustering of Applications with Noise - An advanced clustering algorithm that identifies clusters of varying shapes and sizes from a dataset.

To extend the list of metabolites that serve as biomarkers to differentiate normal, COVID-19, and Post-COVID-19 samples far beyond those identified by linear methods, we implemented some machine learning algorithms onto a public dataset. Figure 1 illustrates a comprehensive workflow of this study’s analytical process, breaking it down into traditional analysis and machine learning approaches. The metabolomics data were obtained from previous reports and are freely available in these references (López-Hernández et al., 2021; López-Hernández et al., 2023b). In summary, selected data comprises 111 identified metabolites across three classes: 142 COVID samples, 48 post-COVID samples, and 38 control samples (See methods: Data). Our analytical workflow is divided into three main branches. In the first one, we combine classical linear and nonlinear dimensionality reduction methods to explore potential features differentiating each clinical group. Dimensional reduction techniques such as PCA (unsupervised), PLS-DA (supervised), and UMAP (unsupervised) are applied to the data in this section.

Additionally, we conducted complementary traditional approaches to identify over-represented markers, particularly differential expression analysis using Earth Mover’s Distance (EMD) and heatmaps of hierarchical clustering using both the raw data and the Z-score standardized data. The second branch is devoted to implementing supervised machine-learning algorithms to classify clinical data. We assessed four classification methods (Logistic Regression, Support Vector Machine, Random Forest, and XGBoost) and selected the best performance. Once we selected the model with the best performance, we carefully and extensively surveyed the importance of the global explainability of each feature through the application of SHAP. The calculation of SHAP values offers a means to interpret the model by assigning a mean weight of feature importance that is not biased by the scale of the data.

The third approach focuses on nonlinear dimensionality reduction and clustering analysis to explore the local explainability of the data. To achieve this goal, we proceeded as follows. Starting from the model with the best performance, we trained it through binary classification between pairs of conditions complemented by a *post hoc* analysis using SHAP values. Afterward, we utilized nonlinear dimensionality reduction via UMAP to elucidate local explanations, providing insights into the formation of subgroups within the high-dimensional SHAP values data. To identify samples containing a set of metabolites with similar classification weights for each subgroup, we applied the Hierarchical Density-Based Spatial Clustering of Applications with Noise (HDBSCAN) algorithm. Having identified each subgroup, the final step involved formulating decision rules for each. To this end, we conducted a multi-class classification of these clusters with XGBoost and obtained their SHAP values. To understand the specific decision rules for each subgroup, we used the dependency plot (SHAP value vs. original magnitude, for example, see Supplementary Figure S1). In the following sections, we present the results obtained for each analysis.

### Limited discrimination by traditional methods in metabolic profiling

**FIGURE 2:**
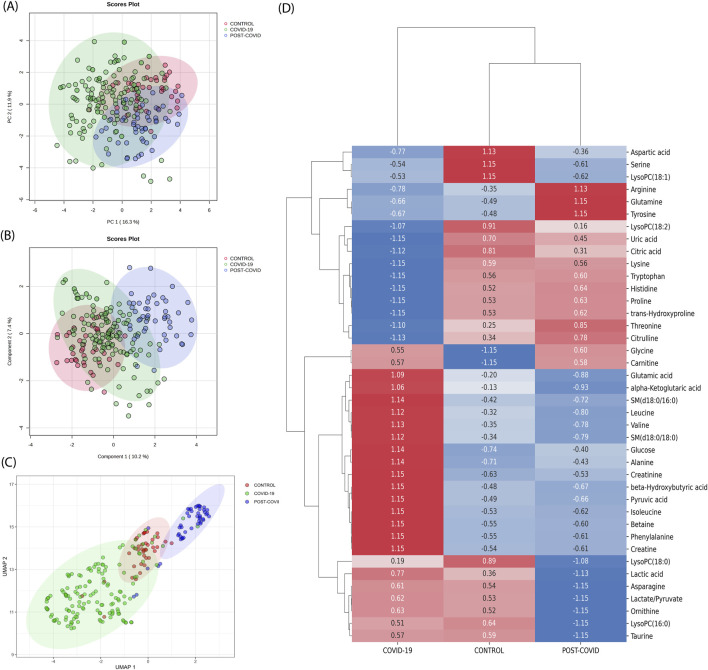
Traditional metabolic Analysis across CONTROL, COVID-19, and Post-COVID-19 States. **(A)** Principal Component Analysis (PCA) scores plot representing the metabolic profiles across samples, with the percentage of variance denoted by PC1 and PC2. **(B)** Partial Least Squares-Discriminant Analysis (PLS-DA) scores plot suggesting the metabolic tendencies of the CONTROL, COVID-19, and Post-COVID-19 groups, with variance represented by Component 1 and Component 2. For PCA and PLS-DA data, median normalization, log transformation, and Pareto scaling were used. **(C)** Uniform Manifold Approximation and Projection (UMAP) visualization of metabolomic data across different conditions. Each point represents an individual sample. The color coding corresponds to the three conditions: CONTROL (green), COVID-19 (red), and POST-COVID-19 (blue). The distinct clustering of samples indicates metabolic differences among the groups **(D)** Heatmap derived from Earth Movers Distance (EMD) analysis illustrating the differential expression of metabolites across the CONTROL, COVID-19, and POST-COVID-19 groups. Displayed values represent the EMD, with positive numbers indicating a higher distribution cost relative to other groups and negative numbers indicating a lower cost. Colors range from blue (lower EMD cost) to red (higher EMD cost), reflecting the magnitude and direction of metabolite level changes among the 40 most expressed metabolites.

Utilizing PCA, the inherent variance within the dataset was initially assessed. As displayed in Figure 2A, the CONTROL, COVID-19, and POST-COVID-19 samples exhibited overlapping regions, emphasizing the complexity of the metabolic patterns using this method alone. Despite PC1 accounting for 16.3% of the variance and PC2 capturing an additional 7.4%, these components did not offer a comprehensive separation of the groups. Similarly, the PLS-DA attempted to maximize the discrimination between the predetermined groups (Figure 2B). While it highlighted some tendencies, the results still showed overlaps, indicating that linear methods, such as PCA and PLS-DA, might not be sufficient to capture the intricate variations present in the metabolic distributions. Different normalization/transformation strategies showed similar trends (Supplementary Figure S2).

On the other hand, we discerned distinct clustering patterns among the three groups (CONTROL, COVID-19, and POST-COVID-19) when one applied UMAP dimensional reduction (Figure 2C). With UMAP, data points representing the COVID-19 group predominantly occupied the lower left quadrant, exhibiting a more dispersed and non-linear distribution. In contrast, the CONTROL group’s data points seemed to concentrate around the center, exhibiting a tighter clustering pattern with sporadic overlap with the group, the POST-COVID-19 group manifested an elongated cluster formation extending towards the upper right quadrant. Notably, while there was some overlap between the COVID-19 and POST-COVID-19 groups, the latter’s data points were distinctly separate from the CONTROL group. This result suggests that non-linear projection could contribute to a better separation of the data.

Finally, with the purpose of comparing our results with those obtained through traditional approaches, we used unsupervised clustering by hierarchical analysis on both the raw and standardized data (Supplementary Figures S5, S6, respectively). In Supplementary Figure S5, as expected based on the raw data, the high magnitude values of glucose and lactic acid metabolites dominate the manifold, serving as the reference point for the Ward method in this clustering approach, and there is no clear clustering of the three phenotypes. In contrast, in Supplementary Figure S6, Z-score scaling of the raw data improves the clustering of the three samples, although it is not sufficient to clearly cluster the structure of the three phenotypes. Neither of these options, raw or standardized z-score data, has a better cluster structure than UMAP. This is due to the Ward method, which assumes that centroid-based algorithms fit well when the data structure is inherently clustered in spherical shapes with Gaussian distributions. Instead, UMAP estimates the local structure of high-dimensional data by constructing a set of local proximity functions that resemble density functions, capturing complicated non-linear shapes of the manifold far beyond just spherical shapes. However, dimensionality reduction methods, including UMAP, are sensitive to high-abundance metabolites, which can overshadow less abundant ones, affecting low-dimensional embeddings. Therefore, the UMAP’s class separation (Figure 2C) might be driven by a few dominant metabolites, such as glucose and lactic acid (Supplementary Figure S5). To enhance the study with respect to dominant variables, we employed differential expression analysis to identify high-abundance metabolites in class distinctions, offering a complementary analysis to dimensionality reduction methods.

### Differential metabolite expression using Earth Mover’s distance (EMD)

Complementing our dimensionality reduction analyses, EMD (one vs. all strategy) was utilized to capture the spectrum of metabolic variations, providing a measure of the distributional shifts between metabolites per condition. EMD revealed distinct patterns of metabolite variations across the three conditions: CONTROL, COVID-19, and POST-COVID-19 (Figure 2D). In the CONTROL group, several metabolites, including aspartic acid, serine, and LysoPC(18:1), were found to be more prevalent, as indicated by the positive EMD values. The COVID-19 group showed that Arginine and glutamine levels exhibited significantly lower levels of arginine and glutamine, which may reflect metabolic disturbances due to the viral infection. Citrulline and threonine also showed reduced levels in this group. In the POST-COVID-19 phase, the metabolite profile did not fully revert to that of the CONTROL group. Some metabolites, like proline and trans-hydroxyproline, approached the baseline levels observed in the CONTROL group, while others, such as glycine and carnitine, remained altered. Several metabolites clustered together in terms of their expression patterns; for instance, lactic acid, leucine, alpha-ketoglutaric acid, and glutamic acid showed a synchronous increase in the COVID-19 group and a subsequent decline in the POST-COVID-19 phase, potentially pointing towards a coordinated metabolic response or shared biochemical pathway. EMD captured metabolic differences that the linear analysis like PCA and PLS-DA, did not detect, it revealed distributional differences between conditions that informed on metabolites overlooked by linear analyses (as shown in the limited intersections in Supplementary Figure S3).

Although the EMD matches dissimilarity between the metabolome distributions between groups ignoring if there is a linear or nonlinear dependency, it relies on the magnitude and dispersion on the metabolome distribution. This approach highlights the hidden information beneath the linear dependence space in which the PCA and PLS-DA stay. Moreover, to address class disparities that are not discernible through conventional methodologies sensitive to magnitude, it is imperative to integrate additional analytical strategies that are not magnitude-sensitive, such as ML approaches.

### Evaluation of multiclass machine learning models and XAI

**FIGURE 3:**
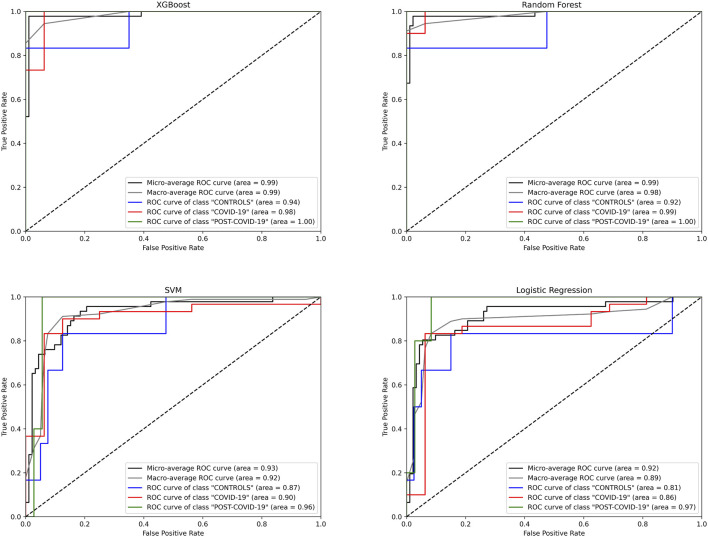
Receiver Operating Characteristic (ROC) curves for different machine learning models predicting three classes: “CONTROLs”, “COVID-19”, and “POST-COVID-19”. Four algorithms were evaluated: XGBoost, Random Forest, SVM, and Logistic Regression. The curves depict the true positive rate (Sensitivity) against the false positive rate (1-Specificity) for each class. The diagonal dashed line represents the line of no discrimination. AUC (Area Under the Curve) values are provided for micro and macro average ROC curves, as well as individual class ROC curves, Blue: CONTROLs, Red: COVID-19, Green: POST-COVID-19.

Despite our preceding analyses were insightful to identify metabolites distinguishing the phenotypes, these methods have the predisposition to emphasize features with higher/lower magnitudes; this can inadvertently overshadow subtler but crucial differences in the metabolites (Evans et al., 2020). Recognizing this limitation, we transitioned to machine learning (ML) models, aiming to harness their ability to predict and classify without unduly favoring dominant features. To this end, we employed 4 different machine learning algorithms, XGBoost, Random Forest (RF), Support Vector Machine (SVM), and Logistic Regression (LogReg), to more precisely identify metabolites whose concentrations can distinguish the physiological stages of the individuals. As shown in Figure 3, the XGBoost model had the highest predictive performance in the ROC curves with a micro and macro Area Under the Curve (AUC) of 0.99 over the other ML models (Table 1).

**TABLE 1:** Performance metrics of machine learning multiclass models. The mean and standard deviation of a variety of metrics of performance of each model are evaluated through cross-validation.

	Train set + cross validation				Test set					
ML method	Accuracy	Precision	Recall	F1 score	Accuracy	Precision	Recall	F1 score	Micro AUC	Macro AUC
XGBoost	0.907 ± 0.059	0.920 ± 0.070	0.879 ± 0.073	0.885 ± 0.067	0.978	0.989	0.944	0.964	0.99	0.99
RF	0.891 ± 0.053	0.905 ± 0.077	0.848 ± 0.073	0.854 ± 0.067	0.957	0.979	0.911	0.941	0.99	0.98
SVM	0.819 ± 0.039	0.793 ± 0.042	0.785 ± 0.056	0.782 ± 0.045	0.804	0.716	0.7	0.707	0.93	0.92
LogReg	0.852 ± 0.071	0.867 ± 0.065	0.806 ± 0.106	0.808 ± 0.097	0.804	0.731	0.767	0.744	0.92	0.89

**FIGURE 4:**
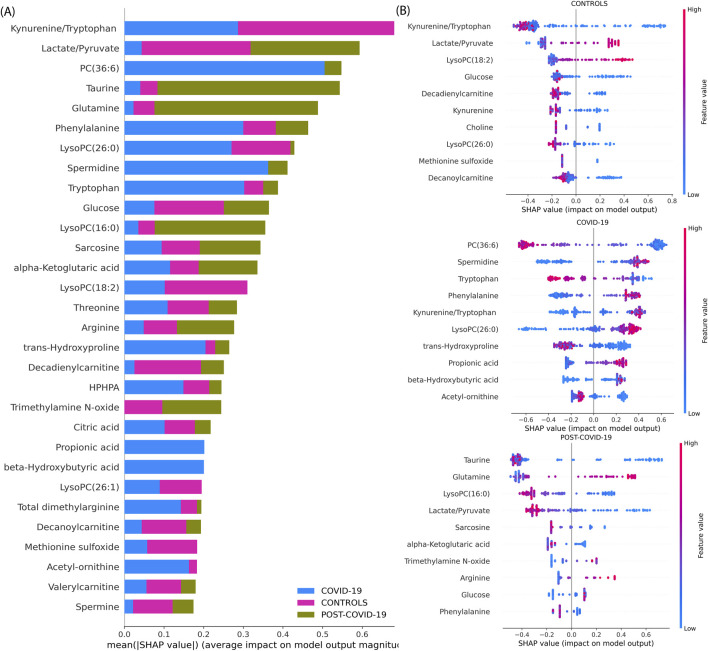
SHAP value analysis of an XGBoost model trained on metabolite data for multiclass classification. **(A)** Average magnitude of SHAP values for each metabolite, indicating their overall importance in the model (Global explainability). **(B)** Dot plots representing individual SHAP values (Local explainability) for each comparison following the Local explainability uses a ‘one vs. all’ approach. For instance, at the top we show the Global explainability of the CONTROLS vs. the rest of the samples (COVID-19 and POST-COVID-19). Colors denote the relative concentration of metabolites, with blue indicating low and pink indicating high concentrations. For example, positive SHAP values for a sample (indicated by a dot) reflect the importance of a feature in classifying a specific category, such as distinguishing ‘CONTROLS’ from other sample groups, including COVID-19 and POST-COVID-19.”

To describe our best model’s explainability, we explored the XGBoost model’s decision-making process by obtaining its SHAP values. Figure 4 provides a comprehensive SHAP analysis. Figure 4A underscores the overall influence of each metabolite in the model when classifying each physiological group. As the same figure shows, the most important variables to classify the groups are the Kynurenine/Tryptophan and Lactate/Pyruvate ratios, PC(36:6), Taurine, Glutamine, Phenylalanine, LysoPC(26:0), Spermidine, Tryptophan, Glucose, LysoPC(16:0) and Sarcosine emerging as top salient features. In Figure 4B, individual sample-level SHAP values are portrayed across three categories: COVID-19, CONTROLs, and Post-COVID-19. In each category, the XGBoost showed different important metabolites with its SHAP explanations. In CONTROLS, the top metabolites based on the SHAPs are Kynurenine/Tryptophan and the Lactate/Pyruvate ratio. In addition, LysoPC(18:2), Glucose, Decadienylcarnitine, and Kynurenine. For COVID-19, the most influential metabolites to distinguish this class from the other are: PC(36:6), Spermidine, Tryptophan, Phenylalanine, and the Kynurenine/Tryptophan, Lactate/Pyruvate ratios. Lastly, for patients with POST-COVID-19 symptoms, the key metabolites differentiating from the other stages are Taurine, Glutamine, LysoPC(16:0), Lactate/Pyruvate, and Sarcosine.

These findings are supported by a body of research that underscores the importance of some of these metabolites in COVID-19. For instance, Ghini et al. identified significant alterations in metabolites such as Glycine and Glutamine in COVID-19 patients (Ghini et al., 2022). Further, Correia et al. found significant metabolic disturbances, including the Phenylalanine, Tyrosine, Lactate, Tryptophan, which change depending on the disease severity (Correia et al., 2022), similarly Jia et al. found glutamine, glutamate, arginine, ornithine, kynurenine and tryptophan (Jia et al., 2022), Song et al. also found increases in various forms of LysoPCs (Song et al., 2020).

### Metabolomic profiling and binary model interpretation using SHAP values

To better understand how the best machine learning model algorithm (XGBoost) classifies each physiological group and find the most important metabolites that explain the classification, we proceeded to build XGBoost models for all binary classification between pairs of conditions (CONTROLS vs. COVID-19, CONTROLS vs. POST-COVID-19 and COVID-19 vs. POST-COVID-19). This strategy provided insights into the mean weight of feature importance (global explanations), asserting its robustness against data scale biases. Adopting explainable artificial intelligence (XAI) techniques facilitated a more transparent interpretation of our machine learning models.

**FIGURE 5:**
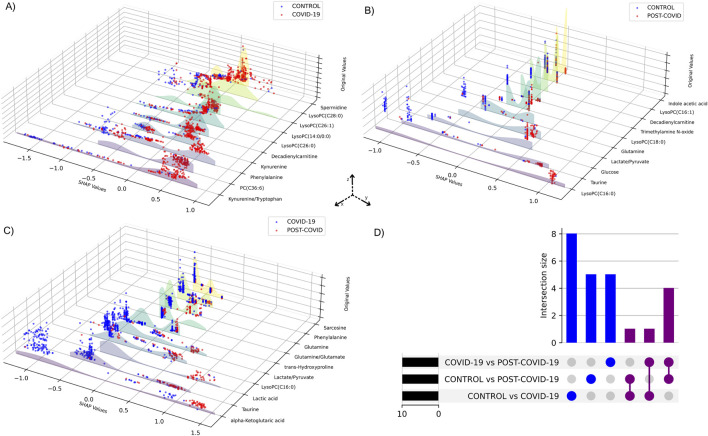
SHAP values of metabolites from binary XGBoost models for distinct group combinations. 3D plots demonstrate SHAP value comparisons between groups: **(A)** CONTROL vs. COVID-19, **(B)** CONTROL vs. POST-COVID-19, and **(C)** COVID-19 vs. POST-COVID-19. Each blue or red dot represents a CONTROL, COVID-19 or POST-COVID-19 according to the respective panel. The y-axis displays the SHAP values, the x-axis ranks the metabolites by their importance to the model and the z-axis shows the metabolites’ original values. Higher SHAP values suggest a greater positive influence on the prediction classification, whereas lower values indicate a negative influence. **(D)** UpSet plot visualizing the intersection of metabolites among three comparisons: CONTROL vs. COVID-19, CONTROL vs. POST-COVID-19, and COVID-19 vs. POST-COVID-19.

Performance metrics of the XGBoost models for each pairwise comparison showed an average of optimal classification similar to the multiclass model (Supplementary Table S1). Figure 5 shows the visualization of the SHAP values derived from binary XGBoost models for the three comparisons. Panel A depicts the SHAP values when comparing CONTROL to COVID-19 samples. Our model suggests that Phenylalanine, the Kynurenine/Tryptophan ratio, and Decadienylcarnitine deploy notable distinctions between the two groups. Similarly, Panel B depicts the SHAP values for CONTROL and POST-COVID-19 samples. In this case, LysoPC(16:0), Glucose, Taurine, and the ratio Lactate/Pyruvate emerge as significant metabolites distinguishing these two groups. Lastly, Panel C compares COVID-19 and POST-COVID-19 samples, revealing metabolites like Glutamine/Glutamate, Taurine, Lactic acid, and alpha-ketoglutaric acid as crucial discriminators. Figure 5 also showed a striking heterogeneity within the metabolic profiles of individuals across the CONTROL, COVID-19, and POST-COVID-19 groups. This heterogeneity is illustrated by the spread and overlap of SHAP value distributions, signifying the varied influence of individual metabolites on the model’s predictions (local explainability). For example, within the CONTROL vs. COVID-19 comparison, the spread of data points in the COVID-19 group across higher SHAP values for metabolites like Phenylalanine and Kynurenine indicates a diverse metabolic response to the infection (Figure 5A). Similarly, within the Control vs. POST-COVID-19 comparison, the POST-COVID-19 group shows a range of SHAP values for metabolites such as Taurine and LysoPC(16:0), reflecting the varied trajectories of metabolic recovery or persisting alterations post-infection (Figure 5B). This metabolic diversity underscores the complex nonlinear relationships and the utility of machine learning models in capturing and interpreting these differences at an individual level, as with the intersections shown in Figure 5D and Supplementary Table S2.

### Metabolic subgroup discovery using explainable embeddings with UMAP and SHAPley values

Building upon the insights gained from the SHAP analysis, which highlighted the specific metabolic influence on our XGBoost model’s predictions and inferable heterogeneity, we explored at a deeper level the metabolic rules that potentially underlie the classes in our data. To achieve a finer-granular explanation of metabolic profiles, we utilized a supervised SHAP-based clustering strategy to define a set of decision rules capable of dissecting the local explainability of the data (Chmiel et al., 2021) (See Methods). As a result, several sub-groups of metabolites with similar contributions in the classification were discerned, facilitating a deeper understanding of disease progression and its metabolic footprint. This rigorous approach culminated in the derivation of sub-group decision rules.

**FIGURE 6:**
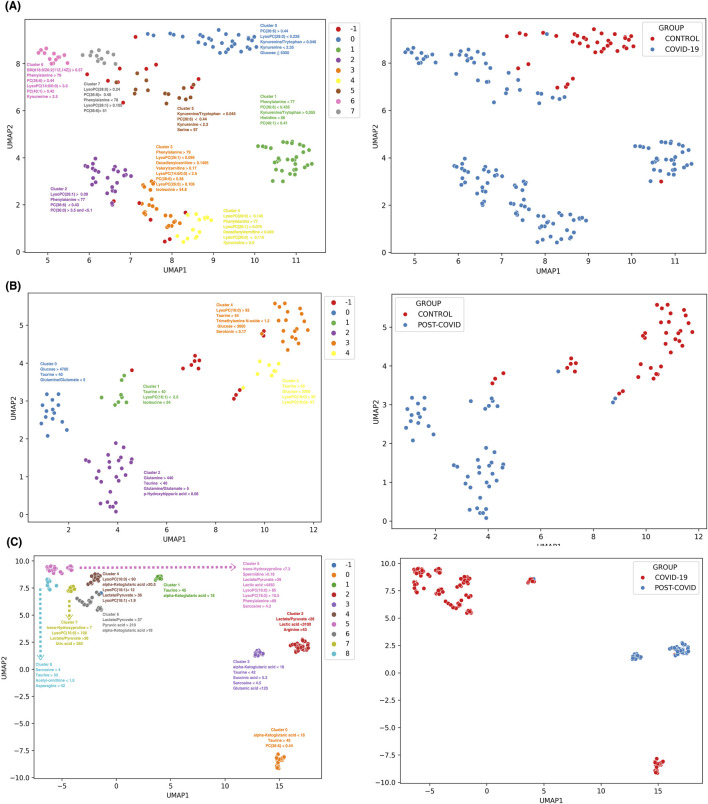
Discrimination and subgroup classification employing UMAP and HDBSCAN on SHAP value distributions from XGBoost models. Each point represents a sample color-coded by distinct metabolic clusters. **(A)** CONTROL vs. COVID-19 with 8 clusters, **(B)** CONTROL VS POST-COVID-19 with 5 clusters, and **(C)** COVID-19 VS POSTCOVID-19 with 9 clusters. Note: Cluster −1 consists of samples that were not dense enough to be classified into a group with HDBSCAN and are classified as noise samples. Clusters are annotated with the most influential features contributing to the subgrouping. The unit of concentration for each numerical value is micromoles as the original data. The right panels show a color-coded binary classification of samples of their original groups.

Figure 6A shows eight discernible metabolic clusters from CONTROL vs. COVID-19. Notably, although specific metabolic markers were predominant in most clusters (such as the kynurenine/tryptophan ratio, kynurenine, and phenylalanine levels, initially identified in our multiclass machine learning model), each cluster features a characteristics combination and concentration of metabolites. CONTROL vs. POST-COVID-19 showed five metabolic clusters (Figure 6B). These clusters showed distinct markers, with some showing pronounced levels of taurine and glucose concentrations, which emphasized the importance of these 2 metabolites. Notably, 3 of the 5 clusters were associated with POST-COVID-19, but showed no difference using the number of symptoms (Supplementary Figure S4). For COVID-19 vs. POST-COVID-19 (Figure 6C), we observed nine clusters. Metabolites such as alpha-Ketoglutaric acid, taurine, and the lactate/pyruvate ratio characterized the clusters, notably 3 of the 4 clusters characterized by POST-COVID-19 patients showed greater distance from COVID-19 clusters, indicating the heterogeneity of the disease.

Overall, the combination of UMAP and SHAP values for the interpretability of the data allowed us to draw two main results. First, at higher levels of resolution, there is heterogeneity in the compositions of the samples, even when they are classified in the same clinical group. Second, as expected, each group inside the physiological class has different rules of classification given by concentrations of a few sets of metabolites. These last classification rules contribute to selecting those metabolites for achieving global or local classification with potential application in future studies. Thus, our analysis can analyze the composition of different physiological groups within each of the COVID-19 patient classes and postulate the rules contributing to their metabolic classifications.

## Discussion

In confronting the monumental health crisis posed by COVID-19 and its sequelae, a thorough understanding of its metabolic implications provides valuable information. Our investigation highlights the complex interplay of metabolites during and post-infection by employing a multi-modal analytical approach, encompassing metabolomics and advanced machine learning techniques. Linear methods alone cannot fully parse the complex nature of metabolic dysregulation across CONTROL, COVID-19, and Post-COVID-19 conditions. (van der Maaten et al., 2009). For example, in UMAP and EMD analyses, we observed clearer demarcations between health states; this reinforces that COVID-19’s metabolic disruption cannot be described linearly (Ghojogh et al., 2021). Furthermore, the persistence of certain metabolomic imbalances in the Post-COVID-19 phase underscores the enduring nature of the viral impact, which could potentially inform the etiology of long-lasting symptoms experienced by patients (Liptak et al., 2022).

XGBoost with SHAP explainability avoided the pitfalls of magnitude biases and improved its explicability. The data analysis discussed here offers a refined metabolic landscape, accentuating subtle yet influential metabolites such as PC(36:6) and Taurine across the COVID-19 and Post-COVID-19 states. The emergence of XGBoost’s superior predictive performance, with AUC scores attaining near-perfect metrics, reflects its adeptness at modeling complex, high-dimensional data. This not only validates the algorithm’s application in high-throughput metabolomics data but also demonstrates its potential in clinical settings for evaluating disease trajectories, such as differentiating states of a healthy state or Post-COVID-19, as it has been proven in other diseases (Guan et al., 2023; Roberts et al., 2021; Hogan et al., 2021; Yi et al., 2023; Moore and Bell, 2022; Cao et al., 2023). As a result, we concluded that our approach identified some metabolites reported in the previous analysis (López-Hernández et al., 2023b), but also other metabolites that were not previously determined as important in the classification. This is the case of LysoPC(26:0), PC(36:6), and alpha-Ketoglutaric acid (Supplementary Table S3); the latter has been found that lower levels in COVID-19 patients may have a higher risk of unfavorable outcomes (Sánchez et al., 2023).

By identifying the most influential metabolites in our classifications, SHAP values have highlighted key metabolites that may play crucial roles in the pathogenesis of COVID-19 and Post-COVID-19 syndrome. According to the SHAP values, the disrupted metabolomic profile of acute COVID-19 (see Figures 4B, 5A) is primarily associated with metabolites participating in the immune response and energy metabolism based on our top metabolites found, for example, elevated SHAP values for metabolites such as Kynurenine, a by-product of the tryptophan metabolism pathway, suggest activation of indoleamine 2,3-dioxygenase (IDO) due to inflammation (Kim et al., 2015). Notably, Kynurenine, a metabolite that reflects the general inflammatory status in the body, has been associated as a severity and mortality marker during acute SARS-CoV-2 infection (Lawler et al., 2021; Abdallah et al., 2024). According to our results, Kynurenine has a differential down-production on COVID-19 subjects respect the other groups. It has been hypothesized that the Kynurenine pathway is responsible for some long-term effects of COVID-19 subjects like neuropathogenesis (Dehhaghi et al., 2024). Moreover, our findings suggest the effects are more related to intense transition prior to activation of the pathway, which produces immunosuppressive metabolites with lasting effects.

In the post-COVID-19 phase, SHAP values indicate a distinct shift in metabolite significance, with Taurine and glutamine standing out (see Figures 4B, 5B). Persistently altered levels of these amino acids point towards a sustained immune challenge (Cruzat et al., 2018) or a delayed return to homeostatic metabolic function post-infection. The consistent impact of Taurine, known for its role in bile salt formation and osmoregulation, may also reflect ongoing oxidative stress and or a lack of cellular detoxification (Cruzat et al., 2018; Baliou et al., 2021; Thirupathi et al., 2020; Wang et al., 2020; Singh et al., 2023). Glutamine’s role in supporting immune cell energy requirements could signal a protracted recovery phase where the immune system remains engaged beyond the clearance of the virus (Koufaris and Nicolaidou, 2021; Aydın et al., 2022; Shah et al., 2020). Understanding these sustained metabolic changes is critical for developing post-acute care strategies and could be integral in preventing long-term sequelae often observed in Post-COVID-19 syndrome patients. Our findings reinforce the observation that there are metabolic pathways that remain altered even in the post-recovery phase (López-Hernández et al., 2023a), (López-Hernández et al., 2023b). For instance, persistent fatigue, a hallmark of Long COVID-19 (What Do et al., 2023), may be tied to the disruptions in energy-related metabolites that we observed; in this instance, Taurine supplementation could be used for patients that have lower levels of this metabolite to counter its symptom (Kim et al., 2022).

Our supervised UMAP-SHAP-based clustering strategy (see Figure 6) allowed for the discovery of intricate subgroups beyond traditional analytical capacities by taking into account a low-dimensional topology based on weight for classification instead of magnitude-based methods. For example, PCA is a linear approach biased in magnitude that fails to separate complex data into groups even with different data preprocessing methods (Figure 2; Supplementary Figure S2). Furthermore, PLS-DA, although a linear supervised method, does not achieve separation in its low-dimensional representations of the data (Supplementary Figure S2). Hierarchical clustering (Supplementary Figure S5) shows no complete separation and reveals a dominance of glucose and lactic acid in the high-dimensional topology, also failing to separate the data. While normalizing with z-scores results in a better cluster structure of phenotypes (Supplementary Figure S6), it is not superior to the obtained with UMAP (Figure 2C). However, UMAP, which is based on densities based on Euclidean distances, achieves better separation of phenotypes, although this low-dimensional representation is dominated by certain variables. Thus, we concluded that UMAP-SHAP-based clustering strategy creates a low-dimensional manifold capable of separating phenotype classes. It is without a bias by magnitude and taking into account the importance for classification.

This novel methodological approach eschews simple distance metrics, instead emphasizing the discriminative importance of metabolites as determined by their contribution to the model’s predictive accuracy. This machine-learning analysis reveals the diversity in the metabolic response to SARS-CoV-2 infection and the varied recovery patterns, which are often homogenized in broader analyses. In the comparison between CONTROL and COVID-19 samples, we observed eight distinct metabolic clusters (see Figure 6A). Each subgroup within the COVID-19 group displayed unique metabolic derangements, indicating the possibility of different viral response phenotypes or stages of disease progression. Normally, COVID-19 is classified using the WHO classification, which ranges from asymptomatic to critical illness (Clinical Spectrum), but a more detailed subclassification could be used to improve treatments. The CONTROL vs Post-COVID-19 (see Figure 6B) analysis presented five metabolic clusters with two key metabolites, Taurine and glucose, standing out in their altered levels. The prominence of these metabolites in certain clusters suggests potential pathways that could be investigated for therapeutic interventions. Interestingly, the majority of post-COVID-19-specific clusters did not correlate with the symptomatology (See Supplementary Figure S4), an observation that points to the complex and possibly nonlinear relationship between metabolic alterations and clinical manifestations of post-COVID-19 syndrome. Additionally, we hypothesized that the previous could be due to inter-subject differences within each group that were not controlled or understood. The variation within Post-COVID-19 clusters indicates possible subtypes of long-term sequelae, underlining the need for personalized approaches in managing these patients. A similar strategy has been applied by Cooper et al. (2021) to COVID-19 symptomatology; Cooper identified 16 different clusters of symptoms, emphasizing the complex heterogeneity of the disease and the necessity for individualized therapeutic strategies using a holistic approach. For future studies, it will be essential to closely correlate these metabolic subgroups with clinical outcomes and symptomatology. Prospective studies, including longitudinal sampling and in-depth phenotyping, are needed to confirm the stability and clinical relevance of these metabolic clusters. Moreover, integrating multi-omics data such as genomics, proteomics, and transcriptomics could offer a systems biology perspective, providing a more comprehensive understanding of the pathophysiological mechanisms at play.

Our study, however, is not without limitations. The reliance on two datasets may introduce biases specific to the population sample. Also, metabolic responses are known to be influenced by a variety of factors, including diet, medication, and comorbidities, which were not controlled for in the datasets. Although we could not separate the confusing variables to provide causation of the symptomatology for COVID-19 and long-COVID-19 subjects, the associations found allows us to dig deeper into the metabolome to provide clearance of the non-linear solid relationships within the heterogeneous data. On the other hand, there is a fundamental need to associate the three physiological stages (healthy, COVID-19 and Post-COVID) with diligent metadata compilation so that the models can account for parameters like patient information, clinical variables, and diet. Our results showed rough associations, these are not limited by the methodology but by the availability of the data. We are aware that separation within groups needs to be taken with caution. Nonetheless, there are differences attached to the disease’s clinical evolution. Future research should aim to replicate these findings across diverse cohorts to ensure the generalizability of the metabolic signatures identified for subtyping.

In closing, our investigation offers a robust analytical framework that provides a comprehensive metabolic viewpoint on COVID-19 and its prolonged impact. The application of machine learning models to metabolomics is an approach that holds great promise for elucidating the multifaceted nature of infectious diseases and its long-term consequences.

## Methods

### Data collection

Datasets were sourced from the Mendeley Database at the following URLs:

• Dataset 1 https://data.mendeley.com/datasets/8zfdjsypd8/1
• Dataset 2 https://data.mendeley.com/datasets/7fnt3nfhdv/2.

Lopez et al. employed these datasets in two distinct studies to pinpoint biomarkers and discern metabolic alterations tied to COVID-19 and its post-infection phase (López-Hernández et al., 2021; López-Hernández et al., 2023b). Both investigations utilized an identical method to yield quantitative readings for 111 metabolites in blood plasma, with the omission of carnitine C14:1 in the Post-COVID-19 dataset. To maintain the veracity of the original concentration values, we did not normalize or scale the metabolite data before its use in the machine learning models.

Our contribution is to present new computational strategies to analyze metabolome data and explore new avenues of biological interpretation, particularly starting from the metabolome data reported by Yamilé et al. (López-Hernández et al., 2021; López-Hernández et al., 2023b). In agreement with the source publication, all the clinical studies and data acquisition were approved by an ethics committee and granted for each data set used in this study. More information about the ethical requirements for each study should be directly requested from the corresponding author of the original publications.

### Principal component analysis (PCA), partial least square discriminant analysis (PLS-DA) and UMAP

PCA is a statistical technique that transforms the original variables into a new set of uncorrelated variables known as principal components. These components capture the majority of variance present in the original dataset and in doing so, reveal dominant patterns. PLS-DA, similar in spirit to PCA, is designed to find the direction in the multivariate space that maximizes the separation between classes or groups. It’s particularly suitable for datasets with more variables than observations. PCA and PLS-DA were conducted using the Metaboanalyst 5.0 software (Pang et al., 2021b). During these analyses, we recognized the need for data normalization to mitigate any artifacts, as these techniques perform worse without normalization. As such, we tried multiple normalization procedures to ascertain optimal parameters. The applied normalization techniques encompassed median normalization, log transformation, and Pareto scaling. The specific results of these procedures are showcased in the Supplementary Figure S2. Also we applied UMAP with default parameters to the raw data.

### Differential expression analysis via Earth Mover’s distance (EMD)

EMD offers a way to measure the “distance” between two probability distributions over a region. It can be perceived as the least amount of work needed to transform one distribution into the other. To dissect differential expression in metabolite data, Earth Mover’s Distance (EMD) was used. This method adeptly captures differences in data distributions. The analysis was performed using the “scprep” library in Python, contrasting EMD values across all the datasets. The derived EMD outcomes rendered a ranked inventory of metabolites, underscoring their relative expression shifts. A positive value indicates that transforming the distribution of that metabolite in the group corresponding to the column (COVID-19, CONTROL, Post-COVID-19) into the distributions of the metabolite in the other groups requires more “work.” Conversely, in general, a positive EMD value means that it is elevated compared to the other groups, while a negative value indicates that it has a decreased value.

### Machine learning model implementation

Different machine learning models were used. The array of machine learning algorithms we tapped into were:

•Random Forest (RF): An ensemble method that constructs multiple decision trees during training and outputs the class that is the mode of the classes for classification, or average prediction for regression.
• XGBoost: An optimized gradient boosting library designed to be highly efficient, flexible, and portable.
• Logistic Regression (LR): A regression analysis method suited for prediction of outcome of a categorical dependent variable based on one or more predictor variables.
• Support Vector Machine (SVM): A supervised machine learning algorithm which can be employed for both classification or regression challenges.

Machine learning algorithms were implemented in Google Colab with Python (v. 3.10). Random forest (RF), XGBoost, logistic regression (LR), and support vector machine (SVM) were written using scikit-learn package. To evaluate the performance of the models, the dataset was split into training and testing sets, the training set comprised 80% of the data, while the remaining 20% was allocated for testing.

### Model evaluation

Each model’s efficacy was estimated using a blend of cross-validation and specific evaluation metrics. A 10-fold cross-validation was executed on the training subset, with accuracy, precision, recall, and F1 score computed through the cross_val_score function from scikit-learn. Post cross-validation, models were further appraised on the testing set using the aforementioned metrics. The superior model was identified based on its performance metrics, encapsulated by:

$$Accuracy=\frac{TP+TN}{TP+TN+FP+FN}$$

$$Precision=\frac{TP}{TP+FP}$$

$$Recall=\frac{TP}{TP+FN}$$

$$F1\;Score=2\times \frac{Precision\times Recall}{Precision+Recall}$$

### Hyperparameter tuning

In the domain of machine learning, the enhancement of model performance is frequently achieved through a meticulous process termed as hyperparameter optimization. For our research, the optimization strategy employed was using a combination of randomized search and cross-validation methodologies. Randomized search, distinct from the exhaustive nature of grid search, offers an efficient exploration of the hyperparameter space by examining a random subset of possible parameter values, leading to faster convergence to optimal values. Complementing this, cross-validation ensured that the model’s evaluation was robust and unbiased by systematically partitioning the dataset into training and validation subsets. For specificity, the hyperparameters scrutinized for each predictive algorithm were:

1. Random Forest (RF):
  ○ n_estimators: Reflecting the count of trees in the forest, which determines the ensemble’s complexity and predictive capability.
  ○ max_depth: Signifying the maximum number of levels in each decision tree, thereby controlling the depth and potential overfitting.
  ○ min_samples_split: Denoting the minimal count of data points placed in a node before the node is split.
  ○ min_samples_leaf: The minimum number of data points allowed in a leaf node.
2. XGBoost:
  ○ n_estimators: Corresponding to the total count of sequential trees to be modeled.
  ○ max_depth: Dictating how deeply each tree can grow during any boosting round.
  ○ learning_rate: Adjusting the contribution of each tree to the final outcome.
  ○ subsample: The fraction of samples used for fitting the individual base learners.
3. Support Vector Machine (SVM):
  ○ C: Regularization parameter that determines the trade-off between achieving a low margin and ensuring the classifier segments most of the data points correctly.
  ○ kernel: Specifies the type of hyperplane utilized to separate the data.
  ○ gamma: Parameter for non-linear hyperplanes, determining the curve’s fit to the data.
4. Logistic Regression:
  ○ C: Inverse regularization strength, which can prevent potential overfitting.
  ○ penalty: Denoting the norm utilized in the penalization.
  ○ solver: Algorithmic approach employed for optimization problems.

### Shapley values

Shapley Additive exPlanations (SHAP) facilitates local prediction interpretations by ascertaining the importance of each metabolomic feature per sample prediction. As a robust *post hoc* IML method, SHAP extends comprehensive global model insights. Rooted in the cooperative game theory methodology of Shapley values (Lundberg and Lee, 2017; Cooper et al., 2021), SHAP offers a fair approach to apportion rewards within a cooperative game. In this context, the game represents the machine learning model, and the Shapley value fairly describes each metabolomic feature’s contribution to the outcome. We computed the Shapley values via the shapTreeExplainer, using the Python SHAP package.

### Supervised clustering using local explanations and manifold learning

This section was accomplished through four steps (Figure 1, Section Subgroup discovery). First, we select a pairwise comparison and calculate the SHAP values of all metabolites from a XGBoost model. Then, by considering all the local explanations of all metabolites for all the patients (SHAP values matrix), we visualized their topological structure into a low-dimensional space through UMAP. Posteriorly, in this reduced space, we calculated the number of clusters through Hierarchical Density-Based Spatial Clustering of Applications with Noise (HDBSCAN). Finally, having identified the clusters of samples, we trained a multiclass XGBoost model and identified the set of metabolites and their rules to classify each cluster. Then, for the decision rules for each cluster, we used dependence plots to illustrate the concentration of metabolites (original concentration values) versus their corresponding SHAP values, along with clusters. This was done to determine decision rules for operators such as lower than (<), or higher than (>), or its combination (for non-linear interactions) of metabolite concentrations (Supplementary Figure S1). We determined the top variables for each cluster until the cluster of interest showed no clear separation in the dependence plot.

## Data Availability

Original metabolome datasets were obtained from studies by Yamilé et al. (López-Hernández et al., 2021; López-Hernández et al., 2023b) and are available at: Dataset 1: https://data.mendeley.com/datasets/8zfdjsypd8/1 Dataset 2: https://data.mendeley.com/datasets/7fnt3nfhdv/2. All codes used in this study are available at https://github.com/resendislab/POST_COVID_Metabolome_MachineLearning.
