# Supplementary material for: Exploring metabolic anomalies in COVID-19 and post-COVID-19: a machine learning approach with explainable artificial intelligence
Source: Front Mol Biosci. 2024 Sep 9;11:1429281. doi: 10.3389/fmolb.2024.1429281 (PMC11417410; doi:10.3389/fmolb.2024.1429281)
Supplement: Supplementary file 1 [file DataSheet1.pdf]

Supplementary information

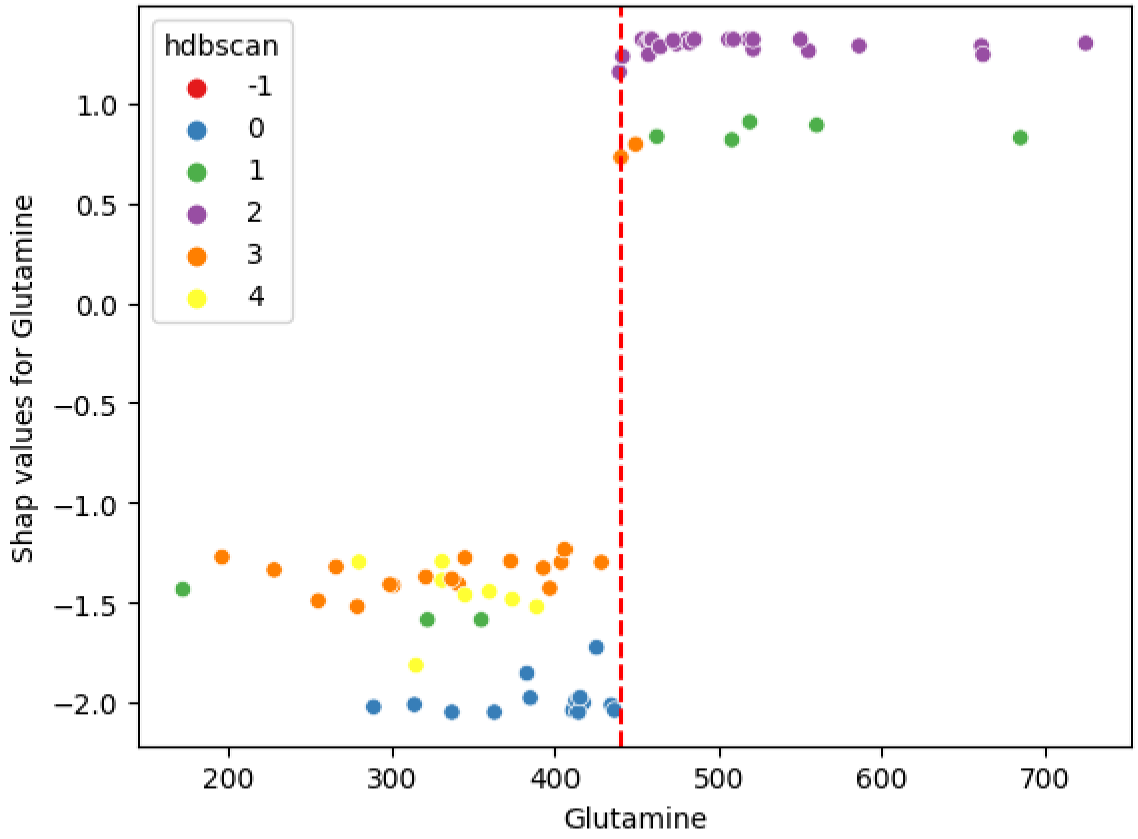

Supplementary Figure 1: Dependency Plot for Cluster 2 in the Control vs. Post-COVID Comparison. The scatter plot displays the relationship between glutamine levels and their corresponding SHAP values for Cluster 2, as identified by HDBSCAN. Each point represents an individual sample, color-coded according to its HDBSCAN subgroup assignment. The vertical dashed red line indicates the threshold level of glutamine used as its decision rules.

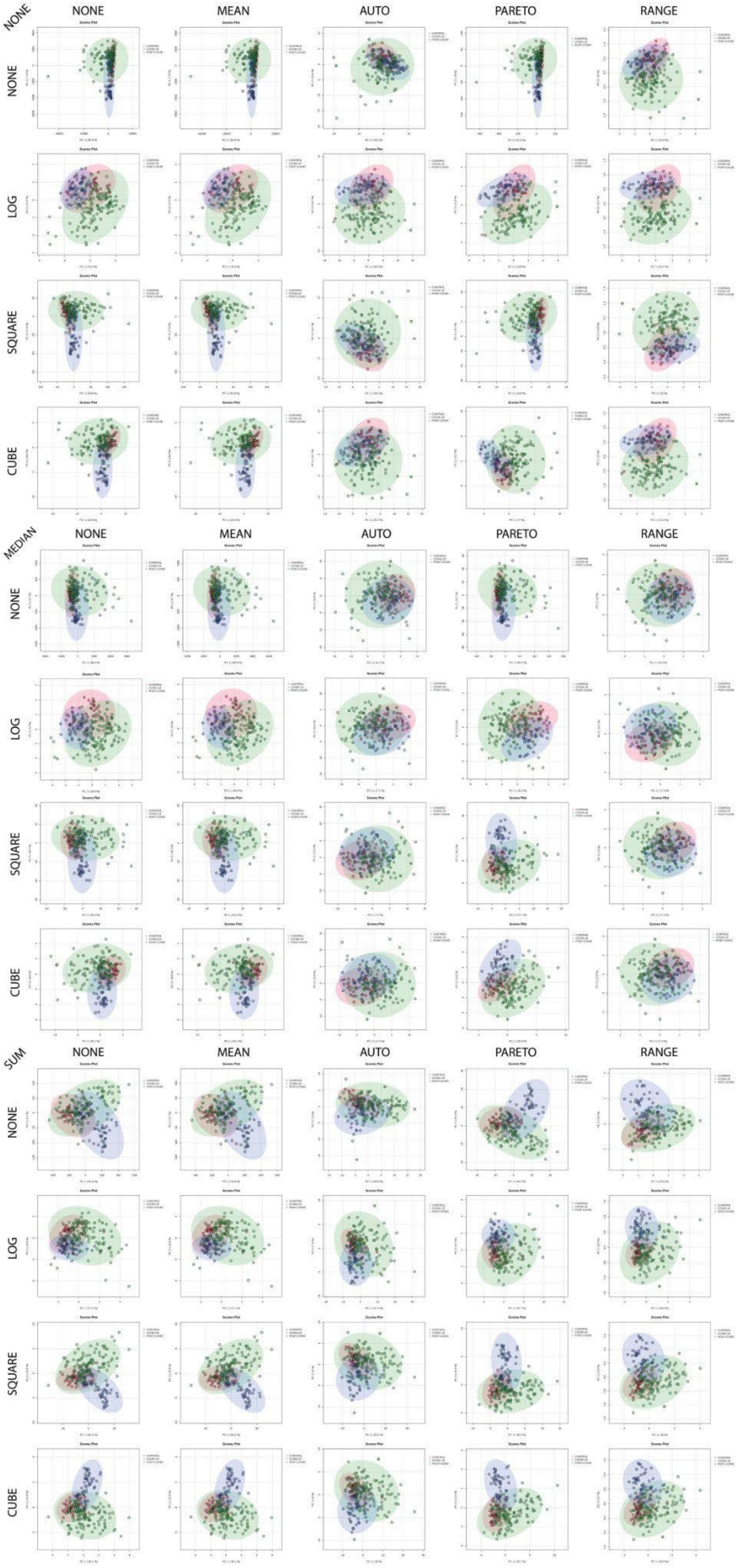
NONE
NONE
MEAN
AUTO
PARETO
RANGE
NONE
LOG
SQUARE
CUBE
MEDIAN
NONE
MEAN
AUTO
PARETO
RANGE
NONE
LOG
SQUARE
CUBE
SUM
NONE
MEAN
AUTO
PARETO
RANGE
NONE
LOG
SQUARE
CUBE

Supplementary Figure 2. Multiple PCA plots using different normalization strategies.

Each panel shows a PCA score plot of the same dataset normalized using a different strategy. The top left corner shows the normalization strategy (None, Sum, Median). The top row shows the different types of scaling (None, Mean, Auto, Pareto, and Range). The left column shows transformation strategies with square root, cube root, and logarithmic transformations.

Supplementary table 1. Performance metrics of machine learning binary models.

			**Cross Validations (CV)**					
**Comparisons**	**AUC**	**Accuracy**	**CV 1**	**CV 2**	**CV 3**	**CV 4**	**CV 5**	**Average CV**
**CONTROL VS COVID-19**	0.991	0.944	0.861	0.889	0.889	0.972	0.917	0.906
**CONTROL vs POST-COVID**	1.000	0.944	1.000	1.000	1.000	1.000	0.882	0.976
**COVID-19 VS POST-COVID**	0.983	0.921	0.947	0.921	1.000	0.974	1.000	0.968

Supplementary table 2. Top 10 features based on SHAP values in the binary machine learning model XGBoost.

**CONTROL vs COVID-19**	**CONTROL vs POST-COVID-19**	**COVID-19 vs POST-COVID-19**
Kynurenine/Tryptophan	LysoPC(16:0)	alpha-Ketoglutaric acid
PC(36:6)	Taurine	Taurine
Phenylalanine	Glucose	Lactic acid
Kynurenine	Lactate/Pyruvate	LysoPC(16:0)
Decadienylcarnitine	Glutamine	Lactate/Pyruvate

LysoPC(26:0)	LysoPC(18:0)	trans-Hydroxyproline
LysoPC(14:0/0:0)	Trimethylamine N-oxide	Glutamine/Glutamate
LysoPC(26:1)	Decadienylcarnitine	Glutamine
LysoPC(28:0)	LysoPC(16:1)	Phenylalanine
Spermidine	Indole acetic acid	Sarcosine

Supplementary table 3. Comparison of metabolite alterations between previously reported and our study.

Metabolite	Previous Study Findings (https://doi.org/10.1038/s41598-023-39049-x)	Our Study Findings (SHAP values)
Glucose	Similar concentration levels in COVID-19 and long COVID-19	Identified as a significant feature for differentiating Controls and post-COVID-19
Uric Acid	Similar concentration levels in COVID-19 and long COVID-19	Not a significant feature in our study
Pyruvic Acid	Higher in long COVID-19 patients compared to healthy Controls	Not a significant feature in our study
Lactate/Pyruvate	Decreased in long COVID-19 patients	Identified as a significant feature for differentiating Controls and post-COVID-19
Kynurenine	Higher in long COVID-19 patients compared to healthy Controls	Identified as a significant feature for differentiating Controls and COVID-19
Tryptophan	Not discussed	Identified as a significant feature for differentiating COVID-19
Kynurenine/Tryptophan Ratio	Increased in long COVID-19 patients	Identified as one of the top features in differentiating control and

		COVID-19 samples
Glutamine	Lower in long COVID-19 patients compared to COVID-19	Identified as a significant feature for differentiating post-COVID-19
Glutamate	Normalized in long COVID-19 patients	Not a significant feature in our study
Glutamine/Glutamate Ratio	Increased in long COVID-19 patients	Significant feature in differentiating COVID-19 from post-COVID-19 states
Phenylalanine	Normalized in long COVID-19 patients	Identified as a significant feature for differentiating COVID-19 and post-COVID-19
Taurine	Lower in long COVID-19 patients compared to COVID-19	Identified as a significant feature in post-COVID-19 classification
Spermidine	Lower in long COVID-19 patients compared to COVID-19	Identified as a significant feature in COVID-19 classification
Alpha-Ketoglutaric Acid	Not discussed	Identified as a significant feature in COVID-19 vs. post-COVID-19 classification
Sarcosine	Lower in long COVID-19 patients compared to controls	Identified as a significant feature in post-COVID-19 classification
Lactic Acid	Increased in COVID-19 phase, normalizing in post-COVID-19	Significant feature identified in COVID-19 vs. post-COVID-19 classification
C10:2	Higher in long COVID-19 patients compared to healthy Controls	Not a significant feature in our study
C18:1	Higher in long COVID-19 patients compared to healthy Controls	Not a significant feature in our study
C10:1	Higher in long COVID-19 patients compared to healthy Controls	Not a significant feature in our study
LysoPC(14:0)	Downregulated in long COVID-19 patients	Identified as a significant feature for differentiating Controls and COVID-19
LysoPC(16:1)	Downregulated in long COVID-19 patients	Identified as a significant feature in COVID-19 vs. post-COVID-19

LysoPC(16:0)	Lower in long COVID-19 patients compared to controls	Identified as a significant feature for differentiating post-COVID-19 samples
LysoPC(18:2)	Similar concentration levels in COVID-19 and long COVID-19	Identified as a significant feature for differentiating Controls
LysoPC(26:0)	Not discussed	Identified as one of the top features in differentiating control and COVID-19 samples
PC(36:6)	Not discussed	Identified as a significant feature for differentiating COVID-19

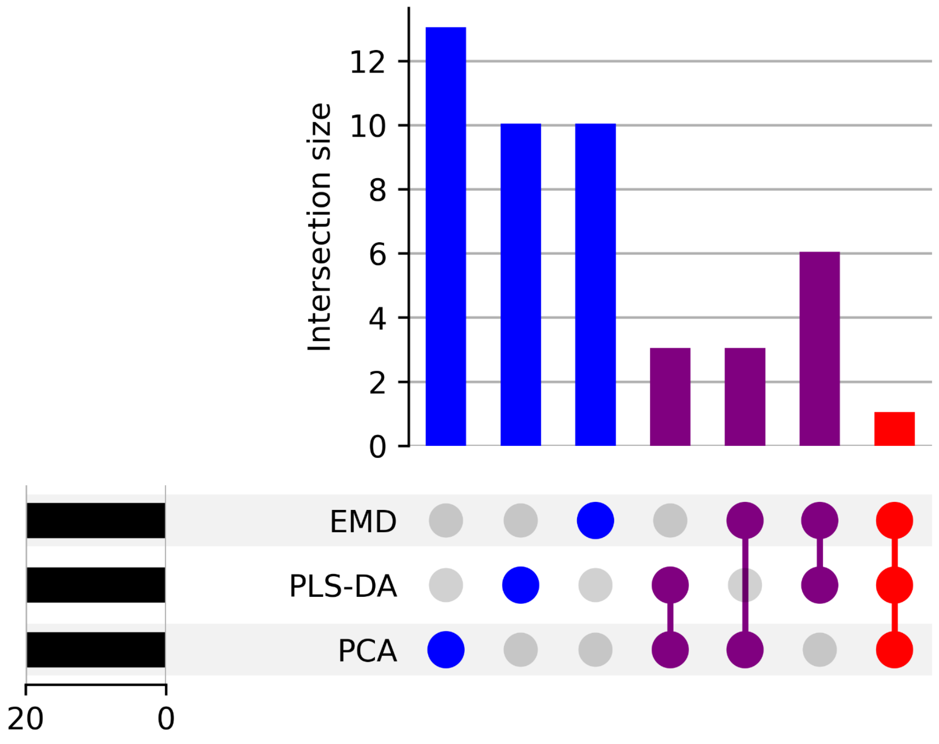

Supplementary Figure 3: UpSet plot illustrating the intersection sizes and relationships of the top 20 metabolites identified by three different statistical methods. Bar chart represents the number of shared metabolites between the methods. The matrix below shows the presence (colored dot) or absence (empty circle) of a particular method's metabolites in each intersection. The methods compared are Principal Component Analysis (PCA), Partial Least Squares-Discriminant Analysis (PLS-DA), and Earth Mover's Distance (EMD).

Metabolites for PCA were selected based on loadings from PC1, for PLS-DA by a Variable Importance in Projection (VIP) score greater than 1.5, and for EMD by their absolute z-score differences. The horizontal bars indicate the number of metabolites identified by each method individually, while the vertical bars represent the size of the intersection sets.

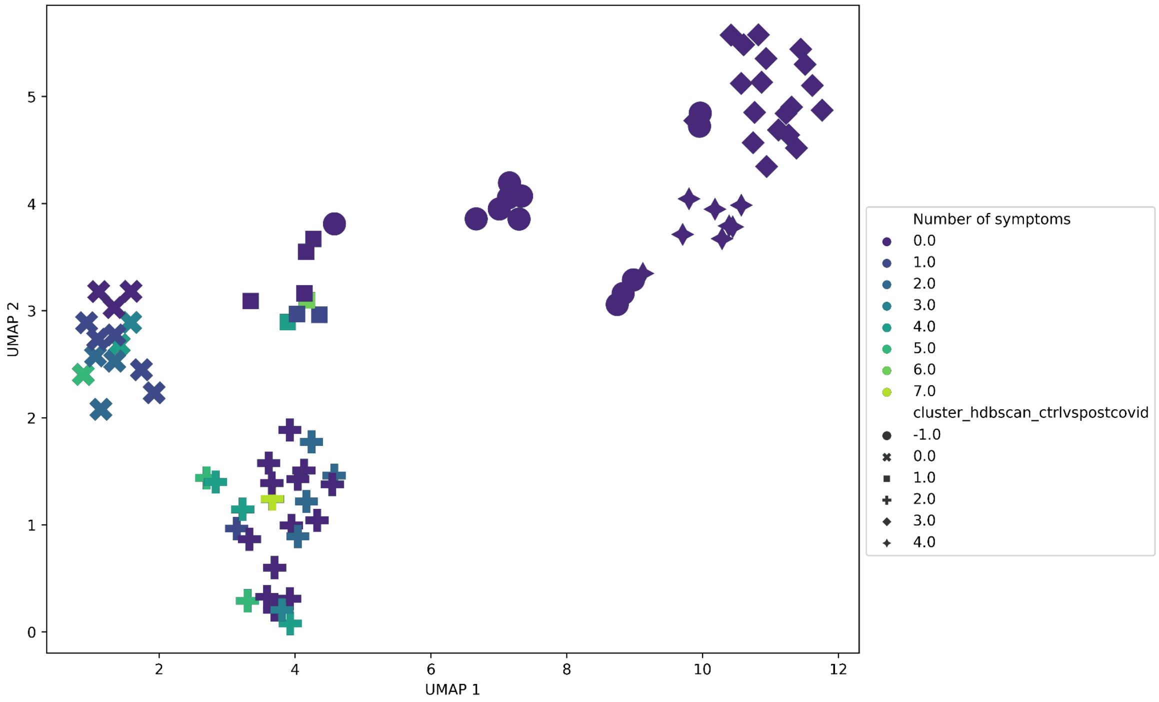

Supplementary Figure 4: Scatter plot of UMAP-based visualization of patients with POST-COVID, colored by the number of reported symptoms and symbol by HDBSCAN cluster number. The UMAP coordinates were generated using SHAP values from a binary machine learning model CONTROL vs POST-COVID.

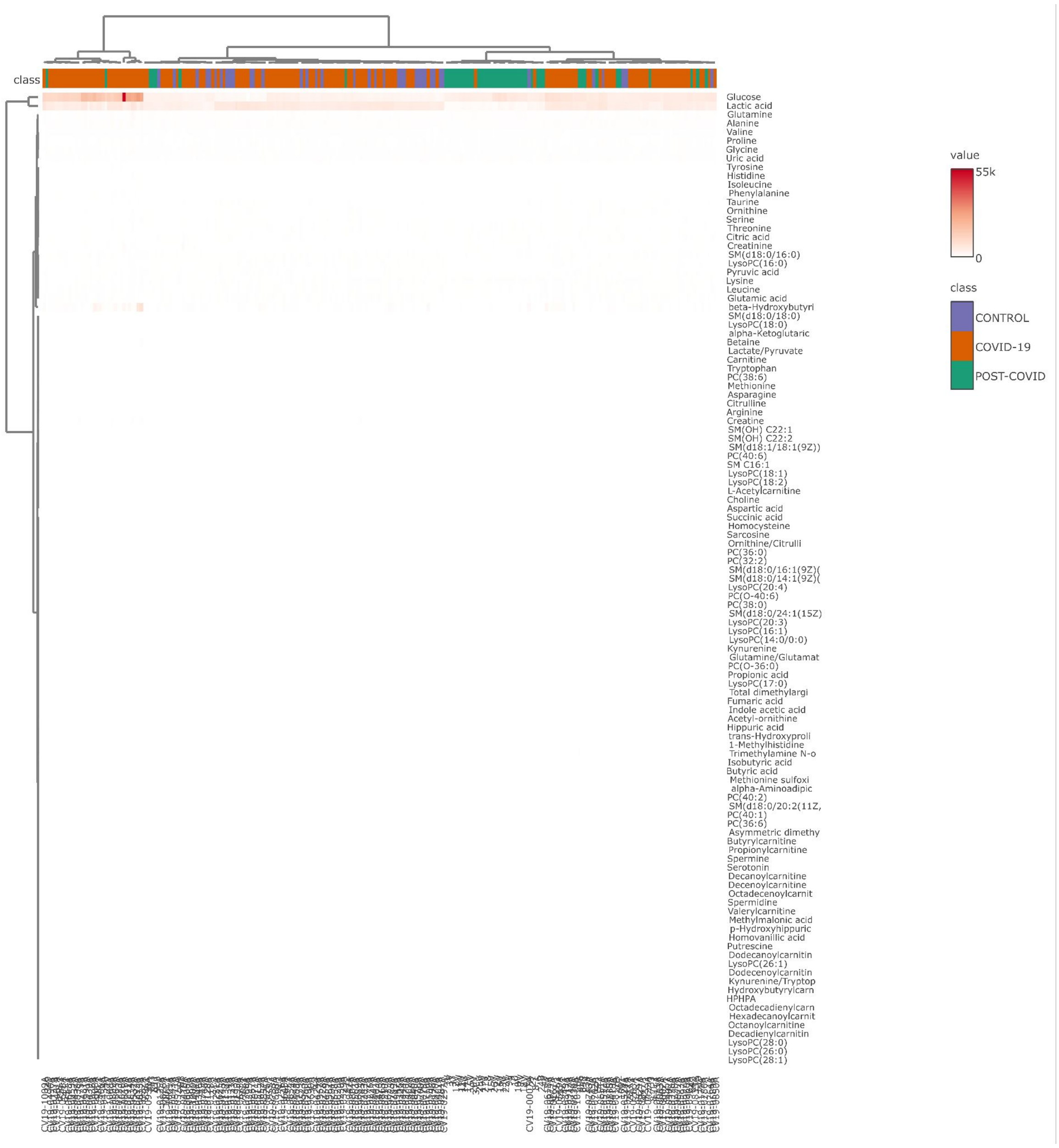

Supplementary Figure 5: Hierarchical clustering from raw data

Hierarchical analysis on the raw matrix. The parameters were as follows: Data source: Original data, Standardization: None, Distance measure: Euclidean, Clustering method: Ward, Color contrast: Default. The output was a hierarchical cluster heatmap for samples and metabolites. Note: Glucose and lactic acid metabolites are dominant due to their high magnitude values. Heatmap made with Metaboanalyst 5.0.

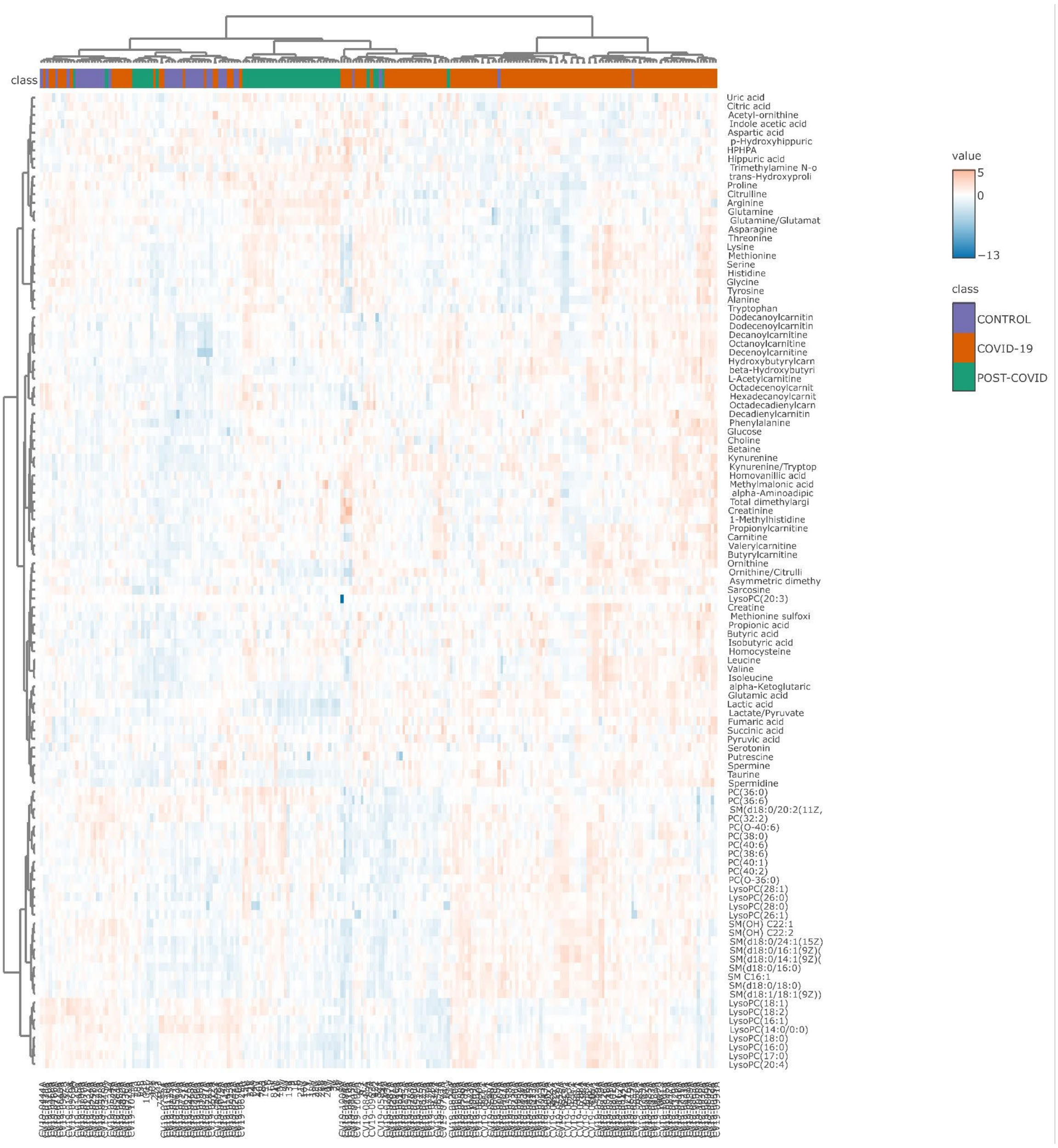

Supplementary Figure 6: Hierarchical clustering with Standardization

Hierarchical analysis on the raw matrix. The parameters were as follows: Data source: Original data, Standardization: Autoscaling, Distance measure: Euclidean, Clustering method: Ward, Color contrast: Default. The output was a hierarchical cluster heatmap for samples and metabolites. Autoscaling is Z score. Heatmap made with Metaboanalyst 5.0.
